# Tandem Short-length Multi-stent Construct for Emergent Revascularization of Occlusive Long-segment Left Middle Cerebral Artery In-stent Stenosis

**DOI:** 10.7759/cureus.7678

**Published:** 2020-04-15

**Authors:** Torin W Karsonovich, Brittany R Bolt, Ajeet Gordhan

**Affiliations:** 1 Neurological Surgery, Advocate BroMenn Medical Center, Normal, USA; 2 Neurology, Advocate BroMenn Medical Center, Normal, USA; 3 Neurointerventional Radiology and Surgery, OSF St. Joseph Medical Center - OSF Healthcare, Bloomington, USA

**Keywords:** in-stent restenosis, wingspan, intracranial stenosis

## Abstract

Endovascular stenting and balloon angioplasty is a feasible although controversial option for intracranial atherosclerotic stenosis refractory to maximize medical management. High rates of symptomatic in-stent restenosis (ISR) have been identified with Wingspan stent (Stryker, Fremont, CA, USA) placement. Revascularization of ISR by way of re-stenting is often attempted, albeit with high risk and low durability. In lesions with long-segment non-focal critical or emergent occluded stenosis, re-stenting with a single balloon mounted stent is not possible due to deliverability of a lengthy device through a tortuous carotid siphon. Tandem drug-eluting stent placement within the middle cerebral artery to address acute, occlusive ISR using a Wingspan stent, with additional stent reconstruction, has not been previously described.

## Introduction

Patients with ischemic symptoms related to high-grade intracranial stenosis (70-99%) have an almost 20% risk of recurrent stroke within one year despite antithrombotic treatment [1]. Studies demonstrate that in the setting of maximal medical therapy, a large cohort of patients will remain symptomatic [2]. Following treatment with Wingspan stent (Stryker, Fremont, CA, USA), approximately 30% of patients experienced in-stent restenosis (ISR) that would require repeat intervention [3]. The re-stenosed segment in more than 50% of cases is more severe in both the length and degree of stenosis [4]. We present a case of a progressive long-segment left middle cerebral artery (MCA) ISR treated with an emergent rescue implantation of tandem drug-eluting stents (DESs) followed by an additional Wingspan stent placement, resulting in clinical recovery and sustained patency of vasculature at six months.

## Case presentation

A 56-year-old male had undergone left MCA stent angioplasty for symptomatic 80% focal stenosis 10 years prior to presentation. Follow-up catheter-based angiography post-stent implantation obtained at six months, as well as one, two, three, and six years demonstrated stable, asymptomatic, and less than 50% ISR at the mid-section of the stent. He presented to the Emergency Department (ED) with transient right-sided hemiparesis and speech difficulty two months after his six-year angiographic follow-up. An MRI of the brain demonstrated multiple acute diminutive watershed infarcts in the left cerebral hemisphere. CT angiography identified an overall diminution in the caliber of the left cervical internal carotid artery (ICA) and branch vascularity of the distal MCA along the convexity distal to the stent. The absence of contrast opacification within the stent suggested intraluminal thrombus or ISR. The patient was admitted to the intensive care unit (ICU), and intravenous heparin was initiated, with subsequent resolution of his symptoms. A P2Y12 assay revealed suboptimal levels of platelet inhibition, and he was then placed on Coumadin®.

He remained asymptomatic for the next four years on warfarin and refused angiography and potential angioplasty. At 10 years, after a transient episode of aphasia, elective outpatient diagnostic angiography was scheduled for a more definitive characterization of the stenosis and potential angioplasty. However, prior to the procedure, the patient presented to the ED with right upper extremity hemiparesis and expressive aphasia. 

**Figure 1:**
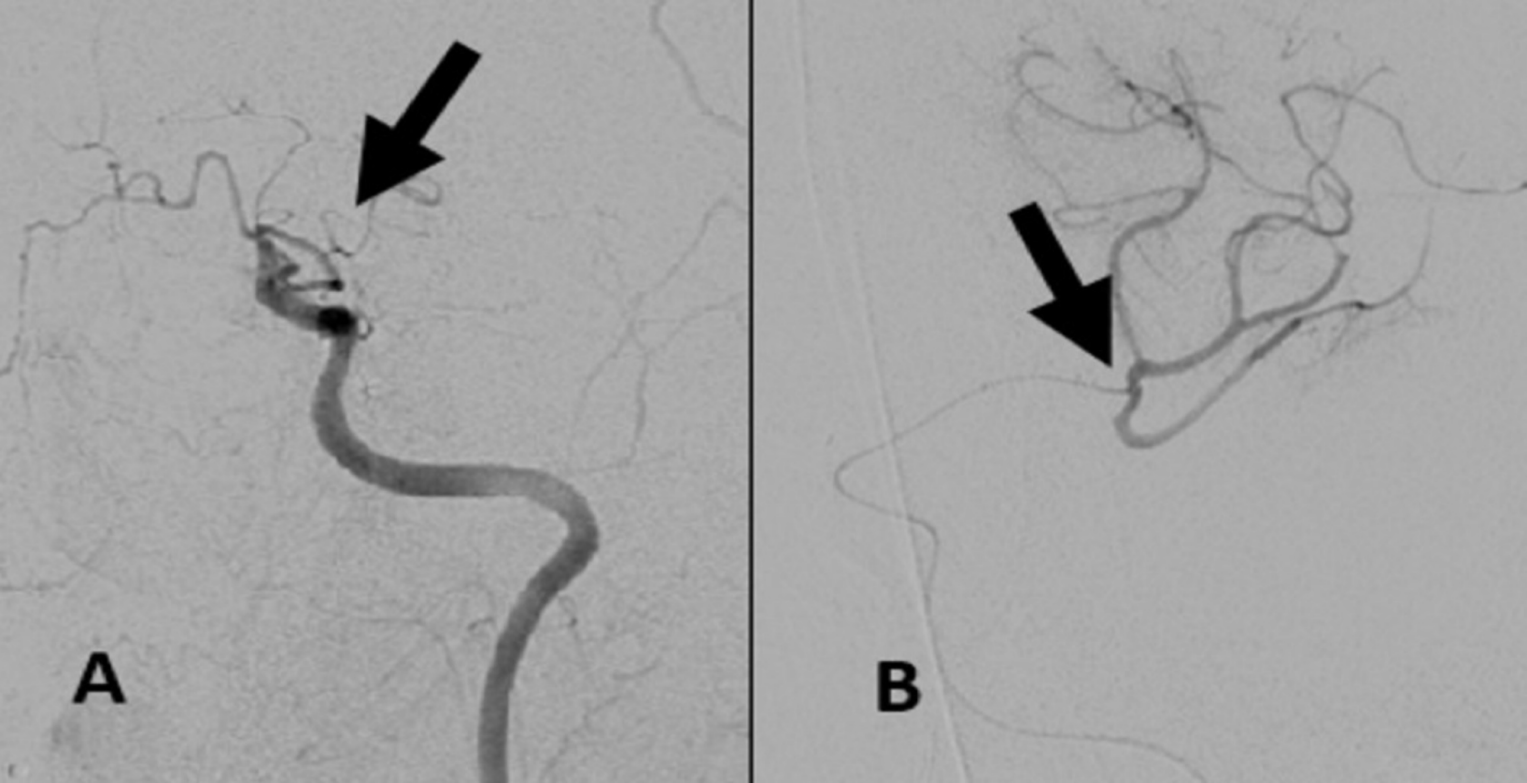
(A) DSA of left ICA revealed complete vessel occlusion distal to the supra-clinoid ICA (black arrow). (B) DSA obtained through the microcatheter positioned within the occluded left MCA revealed patency of the branch vasculature distal to the left MCA stent (black arrow). DSA, digital subtraction angiography; ICA, internal carotid artery; MCA, middle cerebral artery

**Figure 2:**
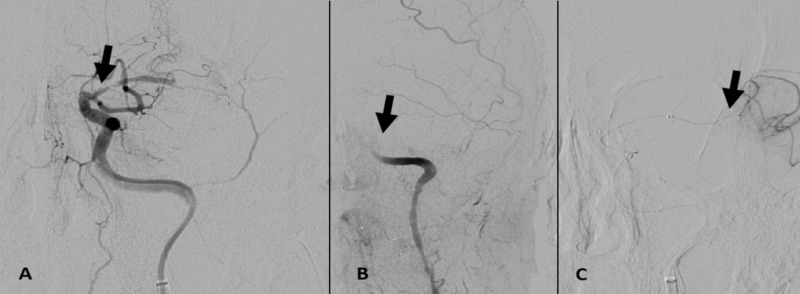
(A) Delayed post-angioplasty DSA of the left ICA identified non-occlusive thrombus within the M1 trunk (black arrow). (B and C) DSA after pharmacologic intervention revealed complete re-occlusion at the level of the supra-clinoid ICA, proximal to the previously placed Wingspan stent (black arrow), with patency of distal branch vasculature (black arrow). DSA, digital subtraction angiography; ICA, internal carotid artery

Emergent diagnostic angiography with intervention was performed under general anesthesia. Digital subtraction angiography (DSA) of the left cranio-cervical circulation using a conventional 5F diagnostic catheter through the right common femoral artery access revealed complete vessel occlusion distal to the supra-clinoid intracranial ICA (Figure 1A). An Excelsior SL 10 microcatheter (Stryker) in tandem with a Transcend 14 (Stryker) exchange length micro-guidewire that was coaxially placed within a Navien 058 (Medtronic, Minneapolis, MN, USA) intermediate guide-catheter was advanced through the intracranial left ICA and through the occluded previously stented left MCA. DSA obtained through the microcatheter revealed patency of the branch vasculature distal to the left MCA stent (Figure 1B). Angioplasty of the left MCA trunk was performed with a Gateway balloon (2.25 mm x 9 mm; Stryker) that was advanced over the exchange-length Transcend 14 mi (Stryker) micro-guidewire. DSA of the intracranial ICA revealed caliber restoration of the left MCA trunk. Delayed post-angioplasty DSA identified non-occlusive thrombus within the M1 trunk (Figure 2A). A bolus dose of eptifibatide was then administered intra-arterially, with a microcatheter placed within the occluded left MCA. This was followed by intra-arterial verapamil and the placement of nitroglycerin paste on the chest. Subsequent angiography of the cervical ICA identified complete occlusion at the level of the supra-clinoid ICA, proximal to the previously placed Wingspan stent (Figure 2B), with patency of the distal branch vasculature (Figure 2C).

**Figure 3:**
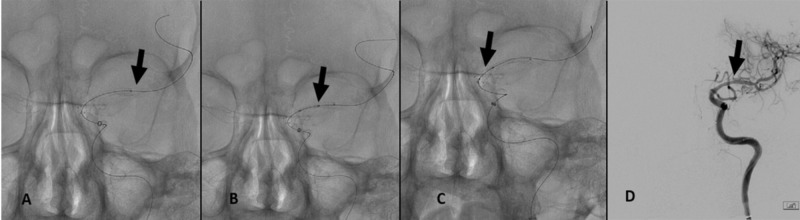
(A) Delivery and deployment of a shorter Xience Alpine (2.25 mm x 8 mm) stent through the carotid siphon into the distal aspect of the MCA (black arrow). (B) Placement of a second Xience Alpine (2.25 mm x 8 mm) stent into the mid-section of the M1 trunk (black arrow). (C) Implantation of a third Xience Alpine (2.75 mm x 8 mm) stent across the proximal aspect of the M1 trunk to cover the entire length of the MCA (black arrow). (D) DSA of the left cranio-cervical circulation demonstrating patency of the supra-clinoid ICA and the M1 trunk, as well as satisfactory filling of distal branch vascularity (black arrow). MCA, middle cerebral artery; DSA, digital subtraction angiography; ICA, internal carotid artery

Advancement of a balloon-mounted coronary Xience Alpine (2.5 mm x 12 mm) (Abbott, Abbott Park, IL, USA) over a retained Mailman micro-guidewire (Stryker) through the left MCA could not be achieved due to difficulty in navigating the relatively long stent through the carotid siphon. Delivery and deployment of a shorter Xience Alpine stent (2.25 mm x 8 mm) through the carotid siphon into the distal aspect of the MCA was achieved (Figure 3A). To address the full length of the MCA, this was followed by placement of a second Xience Alpine stent (2.25 mm x 8 mm) into the mid-section of the M1 trunk (Figure 3B). A third Xience Alpine stent (2.75 mm x 8 mm) was then implanted across the proximal aspect of the M1 trunk to cover the entire length of the MCA (Figure 3C). DSA of the left cranio-cervical circulation demonstrated patency of the supra-clinoid ICA as well as the M1 trunk, with satisfactory filling of distal branch vascularity (Figure 3D).

**Figure 4:**
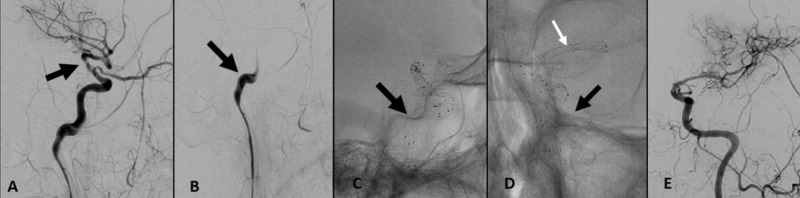
(A and B) Lateral projection delayed DSA through the left ICA after multi-stent placement identified initial stagnation of flow with accumulation of thrombus within the distal left cavernous ICA (black arrow) followed by complete re-occlusion (black arrow). (C) Lateral and (D) frontal projection fluoroscopic spot image with tandem Wingspan stents deployed to cover the extent of the intracranial ICA (black arrows). (E) Post-placement DSA demonstrated patency and satisfactory flow within the left intracranial ICA and left M1 trunk, as well as distal branch vascularity. DSA, digital subtraction angiography; ICA, internal carotid artery; MCA, middle cerebral artery

Delayed DSA identified stagnation of flow with accumulation of thrombus within the distal left cavernous ICA (Figure 4A) followed by complete re-occlusion (Figure 4B). Tandem Wingspan stents were deployed to cover the extent of the intracranial ICA to address a probable stent delivery related dissection (Figures 4B, 4C). Post-placement DSA demonstrated patency and satisfactory flow within the left intracranial ICA and left M1 trunk, as well as distal branch vascularity (Figure 4E).

Post-procedural clinical examination after reversal of endotracheal general anesthesia revealed complete neurologic recovery. A non-contrast head CT identified acute subarachnoid hemorrhage (SAH) within the left Sylvian fissure as well as a small left temporal parenchymal hematoma at the location of a previous subcortical infarct. The patient was managed in the ICU with heparin and aggressive control of blood pressure to prevent reperfusion hemorrhage.

He was discharged home a week later on aspirin 325 mg daily and apixaban 5 mg twice daily. A CT head performed three weeks following discharge revealed a significant reduction in the parenchymal hematoma as well as near-complete resolution of the convexity SAH. At six-month follow-up, he remained neurologically stable, and a catheter-based angiographic study confirmed the patency of the stents within the left intracranial ICA and left MCA.

## Discussion

The challenge of ISR after Wingspan stent placement can often lead to recurrent symptoms of ischemia. In the U.S. Wingspan registry, the incidence of ISR was reported to be 27.9% and was defined as greater than 50% stenosis within or immediately adjacent (within 5mm) to the stent and greater than 20% absolute luminal loss [5,6]. Similar results, a 25% rate of ISR on follow-up angiography, was reported in the National Institutes of Health registry after Wingspan stenting for symptomatic 70-99% stenosis [7]. Derdeyn et al. reported rates of symptomatic ISR of 9.6%, 11.3%, and 14.0% at one, two, and three years, respectively [8]. Yu et al. also reported similar rates of ISR of 10% at one year [9].

Several risk factors for ISR have been identified, including rapid balloon inflation and longer length of stenosis and stenting in the anterior circulation, as well as younger age [10-12]. Turk et al. also reported a higher rate of ISR (66.6% vs. 24.4%) for supra-clinoid lesions compared to other locations [12]. Park et al. reported on 95 consecutive patients who underwent stent angioplasty using Wingspan stents with pre-stent balloon dilation with or without post-stent balloon dilation [13]. They reported a significant difference in the incidence of ISR of 26.3% vs 4.9% (p=0.016) in the pre-stent angioplasty versus the pre-stent and post-stent angioplasty groups, respectively. Furthermore, Yue et al. studied the effects of residual stenosis immediately after stent placement and found that residual stenosis greater than 30% was an independent risk factor for ISR at follow-up [14].

The etiology of ISR is mainly attributed to neointimal proliferation within the lumen of the stent; however, platelet aggregation and stent size can contribute to ISR [15-18]. Lee et al. compared DESs, bare-metal coronary stents (BMSs), and self-expanding stents (SESs) in angioplasty of MCA atherosclerotic stenosis. They reported a reduction of ISR with DESs compared with BMSs and SESs [19]. To combat the consequences of neointimal proliferation, DESs and balloon angioplasty alone have been used in a few cases to treat ISR [9]. However, these methods are not without complications, including dissection, as in our case, and potential worsening occlusive pathology. Additionally, delivery of a single balloon-mounted DES to the MCA is limited by the tortuosity and osseous encasement of the carotid siphon [20].

## Conclusions

Delivery of multiple shorter length balloon-mounted DESs in the MCA allows for navigation through the carotid siphon. Delivery of such stents in the MCA is not without peril, as it risks dissection and progressive re-occlusion. This can be resolved by placing additional Wingspan stents in the carotid siphon. Further studies are needed to assess the feasibility and long-term prognosis of rescue multi-stent intracranial vessel reconstruction intervention.
